# Endoscopic intermuscular dissection of an undetermined submucosal lesion with adaptive traction to obtain a free vertical margin

**DOI:** 10.1055/a-2268-5738

**Published:** 2024-03-01

**Authors:** Louis-Jean Masgnaux, Jean Grimaldi, Valerie Hervieu, Timothée Wallenhorst, Jérôme Rivory, Jérémie Jacques, Mathieu Pioche

**Affiliations:** 1 Gastroenterology and Endoscopy Unit, Edouard Herriot Hospital, Hospices Civils de Lyon, Lyon, France; 2 Histopathology Unit, East Group Hospital, Lyon, France; 3 Gastroenterology and Endoscopy Unit, Pontchaillou University Hospital, Rennes, France; 4 Gastroenterology and Endoscopy Unit, Dupuytren University Hospital, Limoges, France

Endoscopic submucosal dissection (ESD) is a well-established technique for resecting superficial gastrointestinal neoplasms 1 . However, achieving clear vertical margins for submucosal lesions, such as neuroendocrine tumors, can be intricate 2 . The endoscopic intermuscular dissection (EID) method, which involves dissection between the two muscular layers of the rectum, was first developed to treat superficial tumors with vertical extension (T1) 3 . We think this technique could be useful to obtain free vertical margins for submucosal tumors.

We detail the case of a 46-year-old woman who was referred to our center for a 1.5-cm submucosal lesion in the rectum, extending to the anal margin, suggestive of a neuroendocrine tumor ( Fig. 1 ). EID was chosen as the preferred approach given the tumor’s location and potential depth ( Video 1 ).

**Fig. 1:**
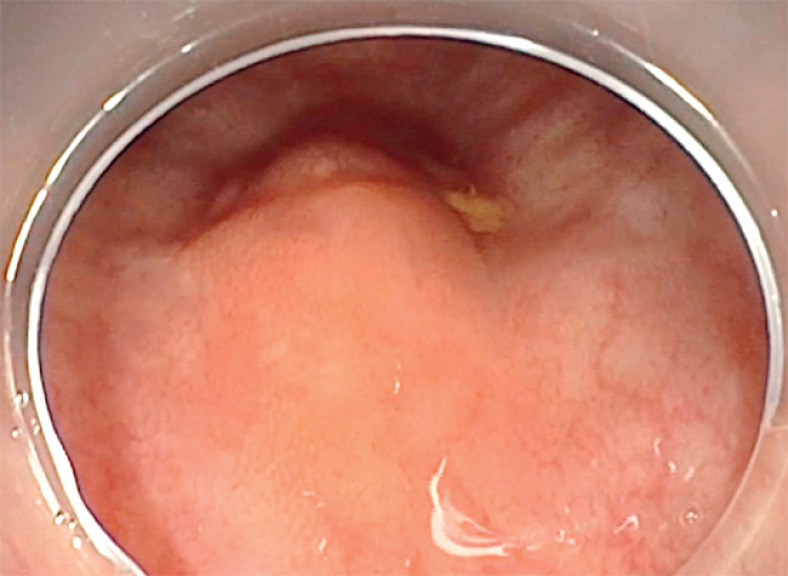
Rectal lesion suspected of being a neuroendocrine tumor.

Intermuscular dissection of an undetermined submucosal lesion with the adaptive traction strategy.Video 1

For effective visualization during EID, the A-TRACT-2+2 adaptive traction device was utilized 4 5 ( Fig. 2 ). The lesion was marked, followed by a circumferential incision. The A-TRACT-2+2 was then positioned ( Fig. 3 ) to provide consistent exposure of the intermuscular space. Its ability to adjust traction ( Fig. 4 ) was beneficial in maintaining a clear view of the dissection plane, reducing the risk of unintended deeper tissue injury.

**Fig. 2:**
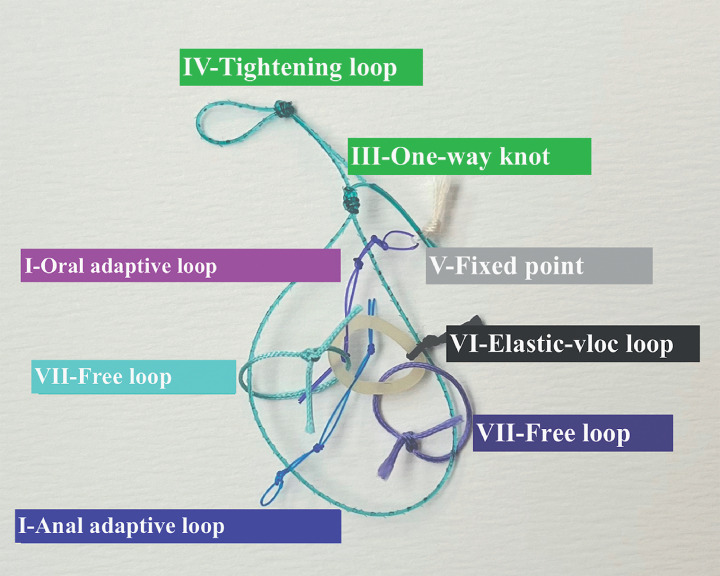
The ATRACT 2+2 adaptive traction device.

**Fig. 3:**
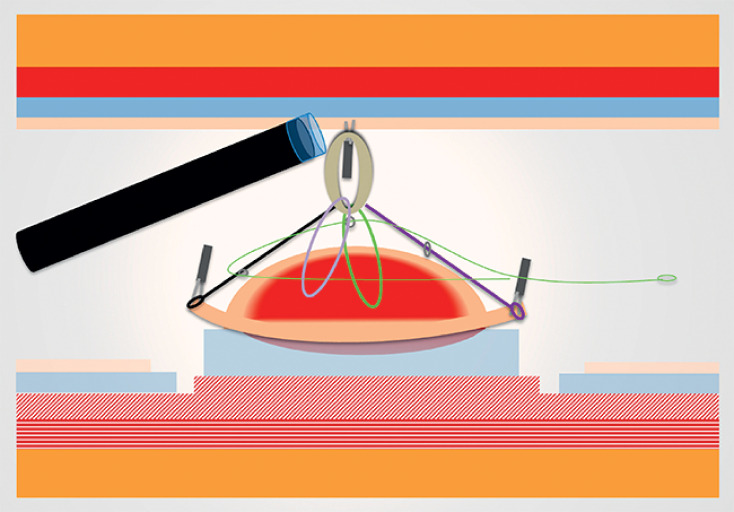
Placement of the ATRACT2+2 device.

**Fig. 4:**
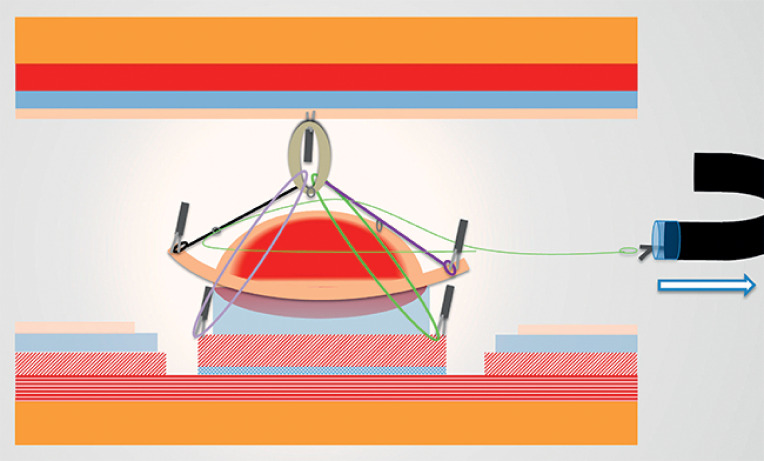
Activation of the device to increase traction and improve intermuscular exposure.

The lesion was resected en bloc. To our surprise, histopathology revealed the specimen was a suppurative granuloma, resected with clear resection margins ( Fig. 5 ).

**Fig. 5:**
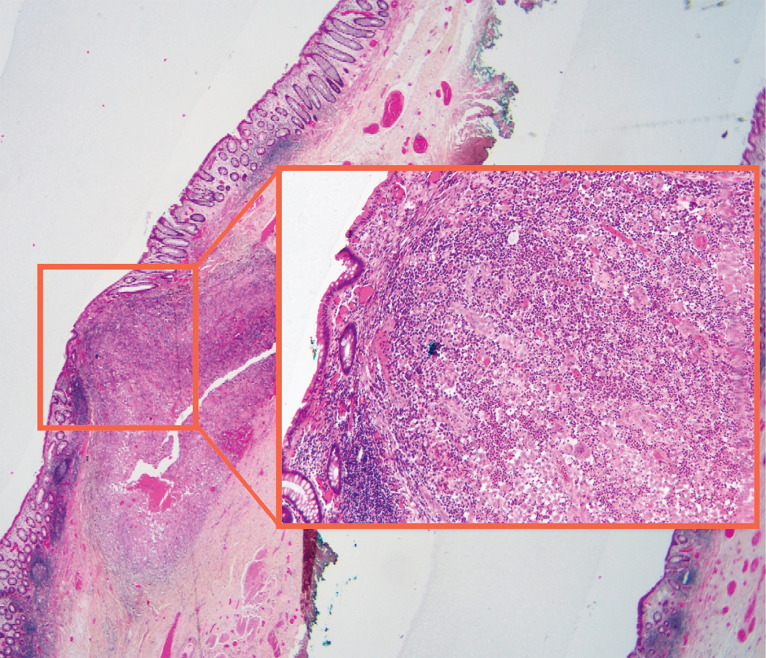
Anatomopathological analysis revealing a suppurated granuloma.

In summary, EID offers a new approach for resecting submucosal lesions suspected of neoplasia. The technique aims to ensure clear resection margins while minimizing potential complications. Proper training and familiarization with the technique and device are essential for optimal outcomes.

Endoscopy_UCTN_Code_TTT_1AQ_2AD

## Notes

Endoscopy E-Videos https://eref.thieme.de/e-videos
*E-Videos* is an open access online section of the journal *Endoscopy* , reporting on interesting cases and new techniques in gastroenterological endoscopy. All papers include a high-quality video and are published with a Creative Commons CC-BY license. Endoscopy E-Videos qualify for HINARI discounts and waivers and eligibility is automatically checked during the submission process. We grant 100% waivers to articles whose corresponding authors are based in Group A countries and 50% waivers to those who are based in Group B countries as classified by Research4Life (see: https://www.research4life.org/access/eligibility/ ). This section has its own submission website at https://mc.manuscriptcentral.com/e-videos .
